# The Effect of Nutrition Education on Glycemic Outcomes in People With Type 1 Diabetes Initiating the Use of Glucose Sensors

**DOI:** 10.1002/edm2.70047

**Published:** 2025-03-23

**Authors:** Vendula Navrátilová, Eliška Zadáková, Jan Šoupal, Jan Škrha, Quoc Dat Do, Lucie Radovnická, Aneta Hásková, Martin Prázný, Eva Horová

**Affiliations:** ^1^ 3rd Department of Internal Medicine, 1st Faculty of Medicine Charles University and General University Hospital in Prague Prague Czech Republic; ^2^ Department of Internal Medicine Masaryk Hospital Ústí nad Labem Czech Republic

**Keywords:** glucose sensors, glycemic outcomes, nutrition education, type 1 diabetes

## Abstract

**Aim:**

To determine whether people with type 1 diabetes (T1D) initiating glucose sensor monitoring experience greater improvements in HbA1c when provided with education on carbohydrate counting and flexible insulin dosing than those who do not receive nutrition education.

**Materials and Methods:**

Our retrospective observational study included 329 people with T1D initiating glucose sensor monitoring between 2015 and 2021. The participants were divided into two groups: one group attended at least one structured educational session with a registered dietitian (*n* = 126), while the other group did not receive structured education (*n* = 203). After 12 months of glucose sensor initiation, we compared glycaemic outcomes and CGM metrics between the two groups.

**Results:**

At glucose sensor initiation, both groups with and without education had similar HbA1c levels (7.64% [60.0 mmol/mol] vs. 7.66% [60.2 mmol/mol]). After twelve months, the education group demonstrated greater improvement in glycemic outcomes (HbA1c 7.17% [54.9mmol/mol] vs. 7.37% [57.1 mmol/mol], *p* < 0.05) and spent significantly more time in the target range than did the group without structured education (68.8% vs. 64.1%, *p* < 0.05). We observed an inverse correlation between the number of completed educational sessions and HbA1c after 12 months, as well as between the number of educational sessions and the change in HbA1c.

**Conclusions:**

People with T1D who initiated glucose sensor monitoring alongside nutrition education showed greater improvements in HbA1c and increased time spent in the target glucose range compared to individuals who did not receive structured education.

**Trail Registration:**

ClinicalTrials.gov identifier: NCT06264271

## Graphical abstract

People with type 1 diabetes who initiated glucose sensor monitoring and received nutrition education on carbohydrate counting and flexible insulin dosing improved their blood sugar compensation more than those without education. This highlights the importance of regular, high‐quality diabetes education.

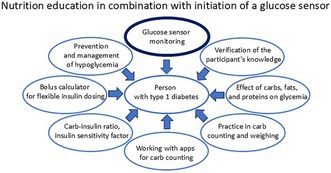

## 1 Introduction

In recent decades, there have been substantial pharmacological and technological advancements in the treatment of type 1 diabetes (T1D). However, one of the most notable changes relates to the overall management approach of this condition. The current trend is to emphasise the importance of intensive structured education immediately following the diagnosis of T1D and to promptly provide individuals with continuous glucose monitoring (CGM) or flash glucose monitoring (FGM). High‐quality education coupled with the use of modern insulin analogues and access to glucose sensor monitoring and other contemporary technologies is the most effective strategy for improving glycaemic outcomes in people with T1D [1, 2].

Education of people with T1D is a long‐term and economically demanding process; however, its efficacy and cost‐effectiveness have been repeatedly demonstrated [3, 4]. Structured education programs on carbohydrate counting [5, 6] and flexible insulin dosing have been shown to enhance glycemic outcomes, improve quality of life, reduce expenses, and achieve these outcomes without increasing the risk of severe hypoglycemia [4, 7, 8]. Furthermore, structured education improves glycemic outcomes while affording individuals greater dietary flexibility, moving away from rigid calorie control and fixed insulin doses [9]. However, significant disparities exist worldwide in access to technology, education, and reimbursement for both.

Another effective way to improve glycemic outcomes and quality of life in people with T1D is to initiate CGM or FGM [10, 11]. Meta‐analyses have demonstrated significant improvements in HbA1c with both monitoring systems [12, 13, 14]. However, the results for FGM alone are not consistent. While FGM has been associated with increased treatment satisfaction and a significant reduction in the incidence of mild hypoglycemia, some studies have not shown significant improvements in HbA1c [15, 16]. Nevertheless, the use of both CGM and FGM significantly increases the time in range 70–180 mg/dL (TIR) [12], reduces the absolute time above range (TAR) and time below range (TBR) [12, 14].

As already mentioned, there is substantial evidence supporting the effectiveness of T1D education programs and the initiation of CGM or FGM in improving glycemic outcomes. However, there is a lack of studies assessing the impact of initiating CGM/FGM alongside structured education. This study aimed to determine whether people with T1D initiating CGM/FGM who received structured education on carbohydrate counting and flexible insulin dosing exhibited greater improvements in glycemic outcomes, as measured by HbA1c and TIR, than individuals initiating glucose sensor monitoring without structured nutrition education. This study also aimed to evaluate other CGM metrics and changes in body weight, BMI, and insulin doses 1 year after glucose sensor initiation in both groups with and without education.

## 2 Materials and Methods

### 2.1 Study Design

This retrospective, observational, and non‐interventional study was conducted at the Diabetes Centre of the 3rd Department of Internal Medicine, General University Hospital in Prague, Czech Republic. Participants were selected from the electronic medical records database. Initially, a basic cohort of all people with T1D attending the Diabetes Centre between January 1, 2015, and January 1, 2021 (*n* = 908) was created. After inspecting the clinical data of each participant, an analysis cohort (*n* = 329) was established and comprised individuals who met the eligibility criteria. The study protocol was approved by the independent ethics review board (Ethics Committee of the General University Hospital, Prague, Protocol No. 89/21 S‐IV), and the study was conducted in accordance with the Declaration of Helsinki.

### 2.2 Study Population

The inclusion criteria were age ≥ 18 years, T1D duration for at least 12 months prior to glucose sensor initiation, and all CGM/FGM procedures initiated at the Diabetes Centre of the General University Hospital in Prague. For individuals using FGM, only those performing at least 10 scans per day were included in the study, as lower scan frequency results in suspension of glucose sensor reimbursement by the payers. The requirement of 10 or more scans per day is a condition set by our local insurance company for covering the glucose sensors to ensure their effective use. In both the CGM and FGM groups, glucose sensor usage had to be at least 70% of the time throughout the 12‐month study period in order to capture the relevant impact of glucose monitoring [17]. Participants from the basic cohort (*n* = 908) who met these inclusion criteria were assigned to the analysis cohort (*n* = 329).

Individuals in the study were treated with either multiple daily injection (MDI) therapy or continuous subcutaneous insulin infusion (CSII). In some participants on CSII, a sensor‐augmented pump (SAP) system with a low glucose suspension was used. Automated insulin delivery (AID) systems were not used in our study due to participant recruitment ending on January 1, 2021, when these systems were just beginning to be introduced in the Czech Republic. Additionally, we excluded several individuals who switched to AID before the end of the study period.

All participants used rapid‐acting or ultrarapid‐acting insulin analogues, and in the case of MDI, all participants used basal long‐acting insulin analogues. No participants were treated with human insulins. Insulin dose data were obtained from clinical records, which are updated at every routine visit.

Exclusion criteria included uncertain diabetes type, pregnancy, change or suspension of CGM/FGM during the study period, and unavailability of glucose sensor data.

### 2.3 Structured Education and Participant Stratification

As part of standard care, all people with T1D were offered structured education on carbohydrate counting and flexible insulin dosing by a registered dietitian, independently of the education for initiating glucose sensor monitoring. Attending the nutrition education was recommended by the physician at each visit, but it was voluntary. After each nutritional education session, every individual was automatically offered a further nutrition session at intervals of no more than 6 months or sooner if needed. Structured education sessions were conducted by a qualified dietitian from the Diabetes Centre of the General University Hospital in Prague and defined as an individual session lasting at least 1 h, focusing on carbohydrate counting and flexible insulin dosing in T1D, tailored to individual dietary preferences, preferred carbohydrate intake per day, and total/basal/bolus insulin doses. The content of the nutrition education was based on the participant's level of knowledge and had the following structure: verification of the participant's knowledge (5 min), basic principles of nutrition management for people with T1D (5 min), the effect of carbohydrates, fats, and proteins on glycemia (5 min), practice in carbohydrate counting and food weighing (10 min), working with applications for carbohydrate counting (10 min), calculation of the carbohydrate‐insulin ratio, insulin sensitivity factor, and use of the bolus calculator for flexible insulin dosing (20 min), and prevention and management of hypoglycemia (5 min). These sessions were required to be documented in written form in the electronic medical records.

**FIGURE 1:**
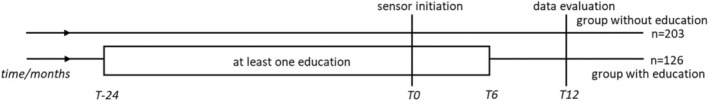
Study profile. Study period: The time from glucose sensor initiation (T0) to month 12 (T12); Nutrition education session(s): Conducted between 24 months (T‐24) before glucose sensor initiation and 6 months (T6) after sensor initiation.

Participants in the analysis cohort (*n* = 329) were divided into two groups based on their participation in nutrition education sessions (Figure 1). Participants who voluntarily attended at least one nutrition education session between 24 months (T‐24) prior to glucose sensor initiation (T0) and 6 months after glucose sensor initiation (T6) were assigned to the group with education. This time period was selected to capture the main impact of education and its significant effect on glycemic outcomes at the time of evaluation (T12), as recommendations suggest that education should be regular and should occur at least once a year to ensure maximum effectiveness [18]. Participants who decided not to participate in the recommended nutrition education were assigned to the group without education. These individuals could receive limited education from their physician during routine visits or engage in self‐education through online resources or educational materials. However, this education was not structured, detailed, or individualised.

### 2.4 CGM and FGM

All participants included in the analysis cohort initiated either CGM or FGM at our Diabetes Centre. The choice of glucose sensor type was based on individual preference, and all participants received standard education for glucose sensor usage. Glucose sensor data were analysed and regularly discussed with individuals during routine visits, and these consultations were documented in written form in the clinical records.

Libre 1 sensors (*n* = 151) (Abbott Laboratories, Abbott Park, IL, USA) with either a Libre reader or the FreeStyle LibreLink smartphone application were exclusively used as FGM. Data from the Libre reader or LibreLink app were downloaded at each routine visit. At the time of the study, FGM technology did not provide alarms.

For CGM, the following glucose sensors were used: Dexcom G5/G6 (Dexcom Inc., San Diego, CA, USA) (*n* = 90), Enlite (*n* = 69)/Guardian Link 3 (*n* = 14) (Medtronic, Dublin, Ireland), and Eversense Senseonics (Senseonics Inc., Germantown, MD, USA) (*n* = 5). Glucose sensor data were downloaded either at routine visits or linked to the Diabetes Centre database through the participant's personal sensor software. The data for the glucose sensor analyses were collected from the corresponding software or clinical records.

### 2.5 Assessments

Demographic and baseline characteristics of the participants in the analysis cohort at the time of glucose sensor initiation (T0) included sex, age, duration of diabetes, HbA1c, body weight, BMI, diabetic complications, insulin doses (total daily, basal, and bolus), type of therapy (MDI or CSII), and type of glucose monitoring (CGM/FGM) (Table 1).

**TABLE 1:** Demographic and baseline characteristics according to allocation into groups with and without structured nutrition education.

	With education (*n* = 126)	Without education (*n* = 203)	*p*
Sex, *n* (%)			
Female	73 (57.9)	103 (50.7)	NS
Male	53 (42.1)	100 (49.3)	NS
Age (SD), years	38.6 (14.7)	39.5 (14.0)	NS
Duration of diabetes (SD), years	14.5 (11.3)	17.8 (11.6)	< 0.01
HbA1c_T0 (SD), %	7.64 (1.10)	7.66 (1.08)	NS
HbA1c_T0 (SD), mmol/mol	60.0 (12.0)	60.2 (11.8)	NS
Body weight (SD), kg	75.4 (15.8)	77.5 (13.7)	NS
BMI (SD), kg/m^2^	25.3 (4.2)	25.6 (4.1)	NS
Complications overall, *n* (%)	34 (27.0)	62 (30.5)	NS
Retinopathy, *n* (%)	25 (19.8)	45 (22.2)	NS
Nephropathy, *n* (%)	5 (4.0)	21 (10.3)	< 0.05
Neuropathy, *n* (%)	13 (10.3)	30 (14.8)	NS
Diabetic foot, *n* (%)	2 (1.6)	1 (0.5)	NS
Insulin dose			
Total daily insulin dose (SD), IU	46.1 (20.6)	48.8 (19.5)	NS
Basal insulin dose (SD), IU	23.2 (10.7)	25.9 (10.6)	< 0.05
Bolus insulin dose (SD), IU	23.2 (13.2)	22.8 (12.0)	NS
Therapy			
MDI, *n* (%)	82 (65.1)	99 (48.8)	< 0.01
CSII, *n* (%)	44 (34.9)	104 (51.2)	< 0.01
Glucose sensors			
CGM, *n* (%)	78 (61.9)	100 (49.3)	< 0.05
FGM, *n* (%)	48 (38.1)	103 (50.7)	< 0.05

Abbreviations: CGM, continuous glucose monitoring; CSII, continuous subcutaneous insulin infusion; FGM, flash glucose monitoring; MDI, multiple‐dose injection; *n*, number of observations or individuals; SD, standard deviation; T0, time of glucose sensor initiation/baseline.

The assessments at month 12 (T12) included HbA1c, body weight, BMI, insulin doses (total daily, basal, and bolus) and changes from baseline. Additionally, the number of education sessions attended, the number of reported episodes of severe hypoglycemia (subject unable to self‐treat), and data from glucose sensors obtained from the 4‐week period prior to evaluation were recorded. These sensor data included the mean glucose concentration, TIR, TBR for levels 1 and 2 hypoglycemia, TAR, and coefficient of variation (CV) [19].

The primary endpoint of this study was the difference in HbA1c between the groups with and without education at month 12 (T12).

The secondary endpoints were as follows:

– differences in TIR (70–180 mg/dL [3.9–10.0 mmol/L]) between groups at month 12,
– differences in TBR percentages in level 1 hypoglycemia (54–69 mg/dL [3.0–3.8 mmol/L]), level 2 hypoglycemia (< 54 mg/dL [< 3.0 mmol/L]), TAR percentages (> 181 mg/dL [> 10.1 mmol/L]), and differences in CV between groups at month 12,
– changes in body weight and BMI from baseline and differences between groups at month 12,
– changes in total daily/basal/bolus insulin doses from baseline and differences between groups at month 12,
– differences in reported episodes of severe hypoglycemia between groups at month 12.

As part of the subgroup analyses, participant demographics and baseline characteristics were statistically compared between the groups with and without education and further evaluated based on glucose sensor type, treatment, and complications. Changes in glycemic outcomes, physical parameters, and insulin doses at month 12 were also evaluated with respect to sensor type, treatment type, and complications. Finally, correlations between the groups with and without education were analysed to explore potential relationships. Changes in HbA1c were tested for correlations with all demographic and baseline characteristics.

### 2.6 Statistical Analysis

Initially, we conducted a power analysis with the following parameters: a significance level of 5%, a power of 80%, and an effect size of 0.5. The primary objective of the study was to compare differences in HbA1c between two groups (with and without structured education) at month 12, and the calculations indicated a minimum sample size of 64 participants in each group, i.e., a total of 128 participants.

Subsequently, we performed several hypothesis tests following the same procedure for each one‐sample or two‐sample test. First, the Shapiro–Wilk test was applied to assess the normality of the distribution for each parameter within each group, with the null hypothesis indicating a normal distribution. For data following a normal distribution, a one‐sample *t*‐test was used to evaluate whether the mean equalled a specified value, using a two‐sided alternative hypothesis. For two‐sample tests, equality of variances between groups was initially tested with the *F*‐test; if variances were equal, a two‐sample *t*‐test was applied, while unequal variances prompted the use of Welch's *t*‐test. When data did not follow a normal distribution, the nonparametric Mann–Whitney test was used for both one‐sample and two‐sample comparisons. The significance level was consistently set at 5%.

Specifically, we used this procedure for two‐sample tests when assessing the equality of mean values of demographic and baseline characteristics between the two monitored groups, with and without structured nutrition education (Table 1). The only exception was for qualitative characteristics (e.g., gender, therapy, type of glucose sensors), where the *Z*‐test for proportions was employed. We applied this procedure for one‐sample tests when evaluating changes within each of the two groups at baseline and 12 months after sensor initiation (Table 2). For comparisons between the two groups 12 months after sensor initiation, we used two‐sample tests (Table 2). Lastly, we also applied these two‐sample tests when conducting separate analyses across subgroups of participants based on CGM and FGM usage, CSII and MDI therapy, and the presence or absence of complications.

**TABLE 2:** The results of the groups with and without structured nutrition education at baseline and 12 months after glucose sensor initiation.

	With education (*n* = 126)			Without education (*n* = 203)			*p* (between groups in T12)
	T0	T12	*p*	T0	T12	*p*	
Education sessions (SD)		4.3 (2.2)			0.0 (0.0)		
HbA1c (SD), %	7.64 (1.10)	7.17 (0.97)	< 0.001	7.66 (1.08)	7.37 (1.11)	< 0.001	< 0.05
HbA1c (SD), mmol/mol	60.0 (12.0)	54.9 (10.7)	< 0.001	60.2 (11.8)	57.1 (12.2)	< 0.001	< 0.05
Body weight (SD), kg	75.4 (15.8)	76.5 (17.1)	< 0.01	77.5 (13.7)	78.5 (14.3)	< 0.001	NS
BMI (SD), kg/m^2^	25.3 (4.2)	25.6 (4.5)	< 0.01	25.6 (4,1)	25.9 (4.3)	< 0.001	NS
Severe hypoglycemia (SD)		0.016 (0.2)			0.015 (0.1)		NS
Glucose sensor							
Glucose (SD), mg/dL		150 (25)			155 (31)		NS
Glucose (SD), mmol/L		8.3 (1.4)			8.6 (1.7)		NS
TIR (SD), %		68.8 (15.3)			64.2 (17.2)		< 0.05
TAR (SD), %		26.3 (15.6)			30.3 (18.5)		NS
TBR_level1 (SD), %		3.77 (2.6)			4.13 (3.3)		NS
TBR_level2 (SD), %		1.17 (1.5)			1.45 (2.5)		NS
CV (SD), %		35.6 (6.5)			36.4 (7.0)		NS

Abbreviations: CV, coefficient of variation; *n*, number of individuals; SD, standard deviation; T0, baseline/time of glucose sensor initiation; T12, 12 months after glucose sensor initiation; TAR, time above range; TBR_level1, time below range in level 1 hypoglycemia; TBR_level2, time below range in level 2 hypoglycemia; TIR, time in range.

We also calculated correlation coefficients between different parameters, using Pearson's coefficient when two continuous variables were assessed, and Spearman's coefficient when examining the relationship between a continuous and a discrete variable.

Data analysis was performed using R version 4.2.1. (2022‐06‐23).

## 3 Results

### 3.1 Demographics and Baseline Characteristics

A total of 329 participants were included in the analysis cohort and divided into a group with education (*n* = 126) and a group without education (*n* = 203) based on whether they had participated in at least one nutrition education session. Overall, participant demographics and baseline characteristics were well balanced between the two groups (Table 1), particularly in terms of mean age and mean baseline HbA1c. The proportion of women was higher in the education group (57.9% vs. 50.7%, not statistically significant), where MDI therapy prevailed (65.1% vs. 48.8%, *p* < 0.01) (while in the group without education, the MDI was balanced with the CSII), and CGM was more prevalent (61.9% vs. 49.3%, *p* < 0.05) (while in the group without education, CGM was balanced with FGM). Participants in the group without education had a longer duration of diabetes (14.5 vs. 17.8 years, *p* < 0.01) and higher doses of basal insulin (23.2 vs. 25.9 IU, *p* < 0.05), while total and bolus insulin doses did not differ significantly between the groups. Almost one‐third of the participants had diabetes complications.

### 3.2 Glycemic Outcomes

Individuals in the education group participated in an average of 4.3 ± 2.2 education sessions from 24 months before to 6 months after glucose sensor initiation. Both groups exhibited significant improvements in HbA1c from glucose sensor initiation to month 12 (Table 2). However, the education group demonstrated a greater improvement in HbA1c than the group without nutrition education, with differences of −0.47 vs. −0.28 percentage points (−5.1 vs. −3.1 mmol/mol), *p* < 0.05.

Subgroup analyses revealed significant changes in HbA1c, weight, and BMI from initiation to month 12, irrespective of sensor type, while treatment type showed an influence. Compared with MDI‐treated participants, CSII‐treated participants (*n* = 148) demonstrated a greater decrease in HbA1c (*n* = 179) (−0.43 vs. −0.30 percentage points; −4.68 vs. −3.23 mmol/mol, respectively), *p* < 0.05. No significant correlations were found between changes in HbA1c and any demographic or baseline characteristics.

The presence of complications also influenced the improvement in HbA1c, and participants who had already developed complications showed greater improvement from initiation (−0.56 percentage points; −6.14 mmol/mol) than participants without complications (−0.27 percentage points; −2.95 mmol/mol), *p* < 0.05.

Additionally, we observed an inverse correlation between the number of education sessions and HbA1c (*r* = −0.12, *p* < 0.05) as well as an inverse correlation between the number of education sessions and the change in HbA1c from initiation to month 12 (*r* = −0.15, *p* < 0.05).

### 3.3 Physical Parameters and Insulin Doses

One of the secondary endpoints was the change in body weight and BMI from glucose sensor initiation to month 12. Body weight increased in both groups, by 1.12 kg in the education group and by 0.97 kg in the group without education. The difference between the groups was not significant. Similarly, BMI increased by 0.33 vs. 0.35 kg/m^2^, with no significant difference between the groups. Changes in body weight and BMI occurred regardless of the monitoring type (CGM/FGM), treatment type (MDI/CSII), or presence of complications.

The study revealed that there were no significant changes in total, basal, or bolus insulin doses from glucose sensor initiation to month 12 in either the group with structured education or the group without education. No influence of the glucose sensor type or treatment type on the insulin dose was observed.

### 3.4 CGM Metrics

The group with a structured education demonstrated a significantly higher TIR than did the group without education (68.8% ± 15.3% vs. 64.2% ± 17.2%, *p* < 0.05) at month 12. In the education group, there was a trend towards a lower percentage of TBR in level 1 hypoglycemia, a lower percentage of TBR in level 2 hypoglycemia, and a lower percentage of TAR than in the group without education, although these differences were not statistically significant (Table 2).

In our subgroup analyses, we examined the differences between CGM and FGM. Regardless of education, we observed significant differences at month 12 in several parameters between participants who used CGM and those who used FGM. Participants using CGM had a greater TIR (68.7% vs. 62.9%, *p* < 0.05), a lower TBR in level 1 hypoglycemia (3.3% vs. 4.8%, *p* < 0.001), a lower TBR in level 2 hypoglycemia (0.9% vs. 1.8%, *p* < 0.05) and a lower CV (35.3% vs. 37.0%, *p* < 0.05), indicating better glycemic outcomes with CGM than with FGM.

In the education group, CGM predominated over FGM, whereas in the group without education, CGM and FGM were balanced. CGM provided better CGM metrics compared to FGM; therefore, we considered whether the resulting difference between the groups in TIR could have been influenced not only by education but also by the choice of sensor. In participants using CGM, there were no statistically significant differences in TIR between the education group and the group without education (Table 3). However, with FGM, education resulted in TIR comparable to CGM, whereas FGM without education led to worsened TIR (Table 3).

**TABLE 3:** Differences in TIR from the perspective of glucose sensor type between the groups with and without structured nutrition education.

Group	*n*	TIR
CGM	178 (54%)	68.7
FGM	151 (46%)	62.9
CGM with education	78 (62%)	68.8
FGM with education	48 (38%)	68.9
CGM without education	100 (49%)	68.7
FGM without education	103 (51%)	60.2

Abbreviations: CGM, continuous glucose monitoring; FGM, flash glucose monitoring; *n*, number of individuals; TIR, time in range.

### 3.5 Severe Hypoglycemia

In both groups with and without education, we observed a very low number of reported severe hypoglycemia events (0.016 vs. 0.015) with no significant difference.

## 4 Discussion

In our study, we demonstrated a significantly greater improvement in glycemic outcomes, as measured by HbA1c, amongst people with T1D who newly initiated CGM/FGM and participated in structured nutrition education on carbohydrate counting and flexible insulin dosing, compared to those who initiated glucose sensor monitoring without receiving nutrition education. When assessing the CGM metrics, we observed that educated individuals had a greater TIR than those who did not receive such education. This secondary endpoint reinforces the primary objective achieved, indicating an improvement in glycemic outcomes in terms of both HbA1c and TIR. These results are consistent with previous research indicating that both structured nutrition education alone [4, 7, 20] and the initiation of CGM or FGM alone [12, 13, 14, 21] contribute to improved glycemic outcomes. In our study, both groups showed significant improvement in HbA1c 12 months after glucose sensor initiation. However, while the improvement in the group without education was likely attributed to the glucose sensor alone, the education group experienced improvement due to the combined effect of the sensor and structured education. Two prospective studies with designs similar to ours evaluated the impact of education on glycemic outcomes. Both studies demonstrated improved HbA1c and TIR in the educated groups; however, the education was focused not on carbohydrate counting and flexible insulin dosing but rather on the use of FGM [22] and rtCGM [23]. Nevertheless, there is a limited number of studies evaluating the effect of structured nutrition education alongside the initiation of glucose sensors. Our study supports previous findings and aligns with current standards of care [11, 18], emphasising the importance of comprehensive management strategies for optimising glycemic outcomes in individuals with T1D.

In subgroup analyses, we observed the impact of diabetes complications on HbA1c improvement. Individuals with existing complications experienced greater improvements in HbA1c than those without complications, irrespective of their participation in structured education. Although we found only a weak correlation between baseline HbA1c levels and improvements in HbA1c (correlation coefficient of −0.35), the relationship between complications and greater HbA1c improvement suggests that pre‐existing complications may primarily be a consequence of historically elevated HbA1c levels. Individuals with pre‐existing complications appear to benefit from glucose sensor initiation, which could improve HbA1c and potentially slow the progression of further complications [24, 25].

The observed inverse correlations between the number of education sessions and HbA1c, as well as the change in HbA1c, may suggest a positive effect of regular nutrition education on glycemic outcomes. The correlations in our study imply that more education sessions could lead to better glycemic outcomes, given that the average number of education sessions was 4.3 sessions over 30 months. However, there is no specific recommended number of education sessions for T1D, as it depends on various factors, such as diabetes duration, treatment targets, complications, physical activity, and life transitions [18]. Moreover, the optimal number of beneficial education sessions is individualised and can also be influenced by factors such as reimbursement and local availability [26, 27].

The increase in body weight and BMI observed at 12 months was significant, but there was no difference between the groups with and without structured education. Interestingly, these increases were independent of the type of glucose monitoring or treatment method, suggesting that external factors such as the COVID‐19 pandemic may have influenced these changes. Notably, despite the increase, there were no changes in the total, basal, or bolus insulin dose at month 12 in either group.

As additional objectives for evaluating CGM metrics, we expected a reduction in the time spent in hypo‐ [28, 29] and hyperglycemia in the education group, attributable to more precise carbohydrate counting and improved capability for flexible insulin dosing [30]. Although our findings showed decreasing trends in the TBR in levels 1 and 2 hypoglycemia and in the TAR in the education group, these differences were not statistically significant.

When comparing the types of monitoring, we observed significant differences between CGM and FGM in TIR, TBR in both level 1 and 2 hypoglycemia, and CV, regardless of education. All these parameters favour CGM, which is consistent with existing evidence [12, 15, 16]. According to the sub‐analysis by sensor type, no significant difference in TIR was observed between the groups with and without nutrition education amongst CGM users. However, amongst FGM users, TIR in the educated group was comparable to that of CGM, whereas in the group without nutrition education, TIR was significantly lower. These results suggest that nutrition education is particularly important for individuals using FGM. While advancements in FGM technology have made its performance comparable to CGM when used with a smartphone application, it is important to note that many individuals still rely on FGM with a Libre reader for monitoring or use applications without alarms. The retrospective design of our study limited our ability to account for the distribution of sensor types between the groups. Furthermore, potential glucose sensor selection bias may exist, as more motivated individuals may have chosen CGM.

We must acknowledge the potential bias that more motivated individuals may have voluntarily sought nutrition education; however, at the beginning of the study (T0), glycemic outcomes based on HbA1c levels were comparable in both groups. We attribute this to the fact that less motivated individuals with unsatisfactory glycemic outcomes also voluntarily attended the nutrition education sessions, likely being encouraged more by their physicians due to their inadequate glycemic outcomes.

Due to the retrospective design, we were unable to completely exclude individuals who may have received education outside of our Diabetes Centre. However, based on the clinical records reviewed, the vast majority of participants (278 out of 329) had been treated at our Centre for more than 24 months prior to glucose sensor initiation, indicating that we captured most of the structured education provided. It is unlikely that individuals attending our Diabetes Centre, who were offered free nutrition education, would seek such education elsewhere. The other 34 participants in the analysis group were referred to our Diabetes Centre by outpatient diabetologists, where limited nutrition education options were available due to a lack of insurance coverage and the fact that specialised dietitians primarily worked in Centres. Nine individuals were newly diagnosed with T1D at our hospital during the 24 months prior to glucose sensor initiation (and met the criterion of having T1D for at least 12 months at the time of initiation) and records of any nutrition education provided are available in the hospital's electronic medical records shared with the Diabetes Centre.

We must also acknowledge the potential bias that participants in the group without nutrition education may have received limited education from their physician or engaged in self‐education. However, such education does not fully correspond to the time span and content of structured nutrition education provided by a qualified dietitian.

Both structured education and glucose sensor initiation are expected to decrease the incidence of severe hypoglycemia. The occurrence of severe hypoglycemia reported in our study was minimal, which explains the lack of significant difference between the groups.

## 5 Conclusions

In conclusion, people with T1D who initiated CGM or FGM together with nutrition education on carbohydrate counting and flexible insulin dosing achieved significantly greater improvements in glycemic outcomes according to HbA1c than individuals who did not receive nutrition education. Additionally, inverse correlations were observed between the number of education sessions and both HbA1c levels and changes in HbA1c. As secondary endpoints, we observed a significantly greater improvement in TIR in the education group, primarily driven by FGM users. Additionally, there were trends towards a lower percentage of TBR in both level 1 and level 2 hypoglycemia, and a lower percentage of TAR in the education group, but these differences were not statistically significant.

These findings support the evidence of the beneficial effect of regular and high‐quality structured nutrition education in people with T1D. Our study provides additional information on the potential improvement in combining the initiation of glucose sensor monitoring with nutrition education. We did not find significant differences in body weight, BMI, or insulin doses between the groups with and without education.

## Author Contributions

The authors take full responsibility for this article.

## Conflicts of Interest

V.N., E.Z., Q.D.D. and A.H. declare no competing interests. J.Š.jr. has received speaker honouraria and consulted for Abbott, Dexcom, Eli Lilly, Medtronic, Novo Nordisk and Roche. J.Š.jr. has received speaker honouraria and consulted for Abbott, Eli Lilly, Novo Nordisk, Sanofi, AstraZeneca, Boehringer Ingelheim and Zentiva. L.R. has received speaker honouraria from Boehringer Ingelheim, Eli Lilly, Sanofi and Novo Nordisk and consulted for Medtronic. M.P. has received speaker honouraria and consulted for Abbott, AstraZeneca, Boehringer Ingelheim, Dexcom, Eli Lilly, Novartis, Novo Nordisk, Medtronic, Sanofi, Takeda and Roche. E.H. has received speaker honouraria and consulted for Eli Lilly, Novo Nordisk, Sanofi, Dexcom and Medtronic.

## Data Availability

The datasets generated during and analysed during the current study are available from the corresponding author upon reasonable request.

## References

[1] R. W. Beck , R. M. Bergenstal , L. M. Laffel , and J. C. Pickup , “Advances in Technology for Management of Type 1 Diabetes,” Lancet 394, no. 10205 (2019): 1265–1273. 31533908 10.1016/S0140-6736(19)31142-0

[2] J. S. Sherwood , S. J. Russell , and M. S. Putman , “New and Emerging Technologies in Type 1 Diabetes,” Endocrinology and Metabolism Clinics of North America 49, no. 4 (2020): 667–678. 33153673 10.1016/j.ecl.2020.07.006 PMC7556222

[3] A. Mauri , S. Schmidt , V. Sosero , et al., “A Structured Therapeutic Education Program for Children and Adolescents With Type 1 Diabetes: An Analysis of the Efficacy of the “Pediatric Education for Diabetes” Project,” Minerva Pediatrica 73, no. 2 (2021): 159–166. 10.23736/S2724-5276.17.04634-5 28176508

[4] DAFNE Study Group , “Training in Flexible, Intensive Insulin Management to Enable Dietary Freedom in People With Type 1 Diabetes: Dose Adjustment for Normal Eating (DAFNE) Randomised Controlled Trial,” BMJ 325 (2002): 746. 12364302 10.1136/bmj.325.7367.746 PMC128375

[5] S. Fu , L. Li , S. Deng , L. Zan , and Z. Liu , “Effectiveness of Advanced Carbohydrate Counting in Type 1 Diabetes Mellitus: A Systematic Review and Meta‐Analysis,” Scientific Reports 14, no. 6 (2016): 37067. 10.1038/srep37067 PMC5107938 27841330

[6] C. E. Builes‐Montano , N. A. Ortiz‐Cano , A. Ramirez‐Rincon , and N. Rojas‐Henao , “Efficacy and Safety of Carbohydrate Counting Versus Other Forms of Dietary Advice in Patients With Type 1 Diabetes Mellitus: A Systematic Review and Meta‐Analysis of Randomised Clinical Trials,” Journal of Human Nutrition and Dietetics 35, no. 6 (2022): 1030–1042. 35436364 10.1111/jhn.13017

[7] A. Shearer , A. Bagust , D. Sanderson , S. Heller , and S. Roberts , “Cost‐Effectiveness of Flexible Intensive Insulin Management to Enable Dietary Freedom in People With Type 1 Diabetes in the UK,” Diabetic Medicine 21, no. 5 (2004): 460–467. 15089791 10.1111/j.1464-5491.2004.01183.x

[8] K. Burghardt , U. A. Müller , N. Müller , et al., “Adequate Structured Inpatient Diabetes Intervention in People With Type 1 Diabetes Improves Metabolic Control and Frequency of Hypoglycaemia,” Experimental and Clinical Endocrinology & Diabetes 128, no. 12 (2020): 796–803. 31091546 10.1055/a-0873-1465

[9] E. C. Vaz , G. J. M. Porfirio , H. R. C. Nunes , and V. D. S. Nunes‐Nogueira , “Effectiveness and Safety of Carbohydrate Counting in the Management of Adult Patients With Type 1 Diabetes Mellitus: A Systematic Review and Meta‐Analysis,” Archives of Endocrinology and Metabolism 62, no. 3 (2018): 337–345. 29791661 10.20945/2359-3997000000045 PMC10118793

[10] N. C. Foster , R. W. Beck , K. M. Miller , et al., “State of Type 1 Diabetes Management and Outcomes From the T1D Exchange in 2016–2018,” Diabetes Technology & Therapeutics 21, no. 2 (2019): 66–72. 30657336 10.1089/dia.2018.0384 PMC7061293

[11] American Diabetes Association Professional Practice Committee , “7. Diabetes Technology: Standards of Care in Diabetes‐2024,” Diabetes Care 47, no. Suppl 1 (2024): 126–144.37922320

[12] M. Elbalshy , J. Haszard , H. Smith , et al., “Effect of Divergent Continuous Glucose Monitoring Technologies on Glycaemic Control in Type 1 Diabetes Mellitus: A Systematic Review and Meta‐Analysis of Randomised Controlled Trials,” Diabetic Medicine 39, no. 8 (2022): e14854. 35441743 10.1111/dme.14854 PMC9542260

[13] I. Dicembrini , C. Cosentino , M. Monami , E. Mannucci , and L. Pala , “Effects of Real‐Time Continuous Glucose Monitoring in Type 1 Diabetes: A Meta‐Analysis of Randomized Controlled Trials,” Acta Diabetologica 58, no. 4 (2021): 401–410. 32789691 10.1007/s00592-020-01589-3

[14] M. I. Maiorino , S. Signoriello , A. Maio , et al., “Effects of Continuous Glucose Monitoring on Metrics of Glycemic Control in Diabetes: A Systematic Review With Meta‐Analysis of Randomized Controlled Trials,” Diabetes Care 43, no. 5 (2020): 1146–1156. 32312858 10.2337/dc19-1459

[15] S. E. Boucher , A. R. Gray , E. J. Wiltshire , et al., “Effect of 6 Months of Flash Glucose Monitoring in Youth With Type 1 Diabetes and High‐Risk Glycemic Control: A Randomized Controlled Trial,” Diabetes Care 43, no. 10 (2020): 2388–2395. 32788281 10.2337/dc20-0613

[16] J. Bolinder , R. Antuna , P. Geelhoed‐Duijvestijn , J. Kröger , and R. Weitgasser , “Novel Glucose‐Sensing Technology and Hypoglycaemia in Type 1 Diabetes: A Multicentre, Non‐Masked, Randomised Controlled Trial,” Lancet 388, no. 10057 (2016): 2254–2263. 27634581 10.1016/S0140-6736(16)31535-5

[17] American Diabetes Association , “6. Glycemic Goals and Hypoglycemia: Standards of Care in Diabetes‐2024,” Diabetes Care 47, no. Suppl 1 (2024): 111–125. 10.2337/dc24-S006 PMC10725808 38078586

[18] American Diabetes Association , “5. Facilitating Positive Health Behaviors and Well‐Being to Improve Health Outcomes: Standards of Care in Diabetes‐2024,” Diabetes Care 47, no. Suppl 1 (2024): 77–110. 10.2337/dc24-S005 PMC10725816 38078584

[19] T. Battelino , C. M. Alexander , S. A. Amiel , et al., “Continuous Glucose Monitoring and Metrics for Clinical Trials: An International Consensus Statement,” Lancet Diabetes and Endocrinology 11, no. 1 (2023): 42–57, 10.1016/S2213-8587(22)00319-9. 36493795

[20] F. Gokosmanoglu and A. Onmez , “Influence of Flexible Insulin Dosing With Carbohydrate Counting Method on Metabolic and Clinical Parameters in Type 1 Diabetes Patients,” Open Access Macedonian Journal of Medical Sciences 6, no. 8 (2018): 1431–1434. 30159070 10.3889/oamjms.2018.256 PMC6108809

[21] J. R. Kluemper , A. Smith , and B. Wobeter , “Diabetes: The Role of Continuous Glucose Monitoring,” Drugs Context 14, no. 11 (2022): 9–13. 10.7573/dic.2021-9-13 PMC9205570 35775072

[22] N. Hermanns , D. Ehrmann , M. Schipfer , J. Kroger , T. Haak , and B. Kulzer , “The Impact of a Structured Education and Treatment Programme (FLASH) for People With Diabetes Using a Flash Sensor‐Based Glucose Monitoring System: Results of a Randomized Controlled Trial,” Diabetes Research and Clinical Practice 150 (2019): 111–121. 30844467 10.1016/j.diabres.2019.03.003

[23] J. H. Yoo , G. Kim , H. J. Lee , K. H. Sim , S. M. Jin , and J. H. Kim , “Effect of Structured Individualized Education on Continuous Glucose Monitoring Use in Poorly Controlled Patients With Type 1 Diabetes: A Randomized Controlled Trial,” Diabetes Research and Clinical Practice 184 (2022): 109209. 35065101 10.1016/j.diabres.2022.109209

[24] A. Skoufalos , R. Thomas , R. Patel , C. Mei , and J. L. Clarke , “Continuous Glucose Monitoring: An Opportunity for Population‐Based Diabetes Management,” Population Health Management 25, no. 5 (2022): 583–591. 36154298 10.1089/pop.2022.0196

[25] M. Yapanis , S. James , M. E. Craig , D. O'Neal , and E. I. Ekinci , “Complications of Diabetes and Metrics of Glycemic Management Derived From Continuous Glucose Monitoring,” Journal of Clinical Endocrinology and Metabolism 107, no. 6 (2022): e2221–e2236. 35094087 10.1210/clinem/dgac034 PMC9113815

[26] D. Christie , R. Thompson , M. Sawtell , et al., “Structured, Intensive Education Maximising Engagement, Motivation and Long‐Term Change for Children and Young People With Diabetes: A Cluster Randomised Controlled Trial With Integral Process and Economic Evaluation—The CASCADE Study,” Health Technology Assessment 18, no. 20 (2014): 1–202. 10.3310/hta18200 PMC4781436 24690402

[27] A. L. McCall , D. C. Lieb , R. Gianchandani , et al., “Management of Individuals With Diabetes at High Risk for Hypoglycemia: An Endocrine Society Clinical Practice Guideline,” Journal of Clinical Endocrinology and Metabolism 108, no. 3 (2023): 529–562. 36477488 10.1210/clinem/dgac596

[28] ISCHIA Study Group , “Prevention of Hypoglycemia by Intermittent‐Scanning Continuous Glucose Monitoring Device Combined With Structured Education in Patients With Type 1 Diabetes Mellitus: A Randomized, Crossover Trial,” Diabetes Research and Clinical Practice 195 (2023): 110147. 36396114 10.1016/j.diabres.2022.110147

[29] S. Suzuki , A. Tone , T. Murata , et al., “Protocol for a Randomized, Crossover Trial to Decrease Time in Hypoglycemia by Combined Intervention of the Usage of Intermittent‐Scanning Continuous Glucose Monitoring Device and the Structured Education Regarding Its Usage: Effect of Intermittent‐Scanning Continuous Glucose Monitoring to Glycemic Control Including Hypoglycemia and Quality of Life of Patients With Type 1 Diabetes Mellitus Study (ISCHIA Study),” Tokai Journal of Experimental and Clinical Medicine 46, no. 2 (2021): 59–68. 34216477

[30] A. Iqbal and S. R. Heller , “The Role of Structured Education in the Management of Hypoglycaemia,” Diabetologia 61, no. 4 (2018): 751–760. 28660491 10.1007/s00125-017-4334-z PMC6448987

